# Cultivar-dependent differences in tuber growth cause increased soil resistance in potato fields

**DOI:** 10.3389/fpls.2023.1095790

**Published:** 2023-06-05

**Authors:** Patrick Skilleter, David Nelson, Ian C. Dodd

**Affiliations:** ^1^ Lancaster Environment Centre, University of Lancaster, Lancaster, United Kingdom; ^2^ Technical Department, Branston Ltd., Lincoln, United Kingdom

**Keywords:** leaf area, root growth, soil compaction, soil moisture, tuber yield

## Abstract

Since soil compaction of potato fields delays shoot emergence and decreases total yield, the causes and effects of this compaction need to be better understood. In a controlled environment trial with young (before tuber initiation) plants, roots of cv. Inca Bella (a *phureja* group cultivar) were more sensitive to increased soil resistance (3.0 MPa) than cv. Maris Piper (a *tuberosum* group cultivar). Such variation was hypothesized to cause yield differences in two field trials, in which compaction treatments were applied after tuber planting. Trial 1 increased initial soil resistance from 0.15 MPa to 0.3 MPa. By the end of the growing season, soil resistance increased three-fold in the upper 20 cm of the soil, but resistance in Maris Piper plots was up to twice that of Inca Bella plots. Maris Piper yield was 60% higher than Inca Bella and independent of soil compaction treatment, whilst compacted soil reduced Inca Bella yield by 30%. Trial 2 increased initial soil resistance from 0.2 MPa to 1.0 MPa. Soil resistance in the compacted treatments increased to similar, cultivar-dependent resistances as trial 1. Maris Piper yield was 12% higher than Inca Bella, but cultivar variation in yield response to compacted soil did not occur. Soil water content, root growth and tuber growth were measured to determine whether these factors could explain cultivar differences in soil resistance. Soil water content was similar between cultivars, thus did not cause soil resistance to vary between cultivars. Root density was insufficient to cause observed increases soil resistance. Finally, differences in soil resistance between cultivars became significant during tuber initiation, and became more pronounced until harvest. Increased tuber biomass volume (yield) of Maris Piper increased estimated mean soil density (and thus soil resistance) more than Inca Bella. This increase seems to depend on initial compaction, as soil resistance did not significantly increase in uncompacted soil. While increased soil resistance caused cultivar-dependent restriction of root density of young plants that was consistent with cultivar variation in yield, tuber growth likely caused cultivar-dependent increases in soil resistance in field trials, which may have further limited Inca Bella yield.

## 1 Introduction

Potato fields worldwide often have levels of soil compaction that limit root growth (Stalham et al., 2007; Johansen et al., 2015a; Sinton et al., 2022). Often, these fields are kept well-irrigated throughout the growing season to avoid soil water deficit and infections from diseases such as common scab (Johansen et al., 2015b), but wet soils are more susceptible to compaction than dry soils (Ghosh and Daigh, 2020). Furthermore, if there is high crop water uptake from the topsoil, but high-volume irrigation events that infiltrate into deeper soil (Reyes-Cabrera et al., 2016), the interface of wet and dry soil layers can be especially sensitive to compaction (Batey, 2009). Agricultural practices are increasingly relying on heavy machinery for field management. This includes seedbed preparation, such as destoning and tilling, as well as planting, irrigation and harvesting, and these machines can easily compact susceptible soil into hard layers. These hard layers typically restrict root growth at depth. This causes roots to become more concentrated in the upper soil layers, exploiting less soil volume, reducing nutrient and water uptake (Johansen et al., 2015a), and reducing yield. As soil compaction can reduce tuber yields by around a third to a half (Van Loon et al., 1985; Stalham et al., 2007; Huntenburg et al., 2021), understanding methods to mitigate this yield loss is vital to close the yield gap.

Soil resistance often increases between planting and harvest in potato (Boone et al., 1978; Huntenburg et al., 2021). There are several possible reasons for this, but likely candidates are changes in soil water content, and production of below-ground biomass. Dry soil has a higher soil resistance than wet soil (Gao et al., 2012), with increased water uptake by developing crops leading to dryer, more resistant soils (Whalley et al., 2006). In rainfed crops, soil resistance is therefore likely to increase through the season as soil moisture is increasingly depleted (Whalley et al., 2006), although in climates where rainfall increases towards harvest, increased soil moisture can reduce soil resistance (Materechera and Mloza-Banda, 1997). Since compaction reduces soil water holding capacity (Torbert and Wood, 1992), compacted soil is more susceptible to soil drying than uncompacted soils, leading to greater increases in soil resistance. Increased below-ground biomass can also compact soils. In soils with a low pore density, which typically occur when a field is destoned (White et al., 2007), roots are forced to create pores which compacts the soil around them (Lucas et al., 2019). Although this compaction can be detected with root diameters as small as 0.25 mm (Lucas et al., 2019), thick roots (diameters > 3.5 cm) can cause soil compaction effects detectable over 4 cm from the root’s surface (Clemente et al., 2005). Although roots of this size typically do not occur in most annual crop plants, including potato (Yamaguchi, 2002), tubers can easily exceed this size, and thus may increase soil resistance.

Cultivar variation in root growth was associated with increased drought tolerance (Puértolas et al., 2014; Boguszewska-Mańkowska et al., 2020), but no variation in compaction tolerance was established in field trials of potato (Van Oijen et al., 1995), possibly because of limited genetic diversity in the cultivars investigated. Nevertheless, cultivars from the *tuberosum* and *phureja* groups, as well as cross-breeds, show wide variation (three-fold differences across 28 cultivars) in root length in field trials ten weeks after emergence, during tuber bulking (Wishart et al., 2013). *Phureja* group cultivars are diploid, whilst *tuberosum* group cultivars are tetraploid (Huamán and Spooner, 2002). *Phureja* group cultivars typically yield 40% less tuber mass per plant than *tuberosum* group cultivars (Wishart et al., 2013), with most cultivars producing a larger leaf area and root number than *tuberosum* group cultivars, although total root system size was similar. However, to our knowledge, the sensitivity of these different groups to soil compaction has not been investigated.

An initial controlled environment experiment identified 2 cultivars from these groups (Maris Piper from the *tuberosum* group and Inca Bella from the *phureja* group) that differed in their sensitivity of root growth to soil compaction. Previous field trials by Branston Ltd. (unpublished) found typical yields of Inca Bella to be 35 t/ha under ideal conditions, with Maris Piper yielding 50 t/ha. Thus, two subsequent field trials grew these cultivars in soils varying in resistance, with soil moisture, root growth, and tuber growth measured to determine their possible impact on soil resistance. The more tolerant cultivar (Maris Piper) was hypothesized to produce a greater root density and leaf area in compacted soil, thereby producing higher tuber yields but potentially compacting the soil to a greater degree. As its root growth is less sensitive to soil compaction, this should promote vegetative development allowing greater water uptake and increased soil drying under rainfed conditions, thereby increasing soil resistance more than in the less tolerant cultivar. This feed-forward response demonstrates the importance of identifying suitable cultivars for planting when high soil resistance is anticipated.

## 2 Materials and methods

### 2.1 Controlled environment (CE) experiment

A sandy-loam topsoil (Norfolk Topsoils, Heavingham, UK) with 71% sand, 26% silt and 3% clay was used. Pots were custom-made from sections of cylindrical PVC pipe (Keyline, Northampton, UK) with an interior diameter of 6.4 cm and a height of 26 cm. Soil was prepared for compaction by being air dried, and passed through a 10 mm sieve. The compacted treatment was produced by adding water to 10% soil water content, and using an arbor press (Model PK 3000, Jack Sealy Ltd., Bury St. Edmunds, UK) with a fitted metal disc that matched the pot diameter using a compacted stress of 4.60 kN/m^2^. Soil was compacted in 2 cm intervals to ensure a constant resistance throughout the pot. Compacted soil was applied until the soil level was 4 cm below the pot’s top, where a potato tuber with a length between 15 mm and 25 mm was placed. Loose soil was produced by compacting the air-dried soil with a force of 1.40 kN/m^2^. Soil resistance was measured using a hand penetrometer (Van Walt, Haslemere, UK) at 5 cm intervals through five pots per treatment. Loose soil had a resistance of 0.3 ± 0.06 MPa (n = 5), whilst resistance in the compacted soil was 3.0 ± 0.16 MPa (n = 5). Loose soil was then applied to cover the tuber for a total soil volume of 772 cm^3^.

The potato cultivars Maris Piper (*tuberosum* group) (TLC potatoes, Banchory, UK) and Inca Bella (*phureja* group) (Branston Ltd., Branston, UK) were grown in either compacted or loose soil, with five replicates of each treatment. Plants were grown in a walk-in CE room with a sixteen-hour day with a 24°C daytime temperature and a night temperature of 16°C with a PPFD at pot height of 450 μmol m^-2^ s^-1^ from metal halide lamps (Osram Powerstar HQI-T, Munich, Germany). Soil mass per pot was determined by subtracting pot weight from the weight of the pot with soil in. Compacted pots had 10% SWC, confirmed using a WET Sensor (Delta-T, Cambridge, UK) and thus the soil mass was divided by 1.1 to obtain dry soil mass. The soil masses were then multiplied by 1.2 to calculate target soil mass when pots were watered. Soil water content was maintained at 20% by watering every 2-3 days. To account for variation in time to break dormancy, plants were harvested four weeks after their first shoot emerged. Leaf area was measured with a leaf area meter (Model LI-3100C, LI-COR, Nebraska, USA), and total root volume per plant was measured using WinRHIZO software (Regent Instruments, Quebec, Canada). Root density per pot was calculated thus: $Root\;density\;\left(cm^{3}/cm^{3}\right)=\frac{Root\;Volume\;\left(cm^{3}\right)}{772}$ , where 772 is the volume of soil per pot in cm^3^.

### 2.2 General methodology

Two field trials were undertaken between May and September in 2021 (trial 1) and 2022 (trial 2). Both experiments utilized two potato cultivars provided by Branston Ltd. (Branston, UK): Maris Piper (*tuberosum* group) and Inca Bella (*phureja* group). As potato crops in the UK are grown in rotation to avoid soilborne pests and diseases, these trials took place on different sites.

Trial 1 took place on a silty loam soil (14% sand, 74% silt and 12% clay) with a previous crop of winter wheat located at a latitude of 53.203188, and a longitude of -0.402652. The trial was not irrigated throughout the growing season. Rainfall and temperature data was logged by an Automatic Weather Station (Sentek, Stepney, Australia). The field received 148 mm of rainfall in May after planting, 92 mm in June, 109 mm in July, 34 mm in August, and 1 mm in September before harvesting. Further weather data can be found in Data Sheet 1. The field was fertilized before planting with 220 kg/ha nitrogen, and 180 kg/ha potassium. Fourteen days before forming beds for planting, the trial area was cultivated to a depth of 25 cm. The trial area was divided using a randomized block pattern with three replicates and four treatments: uncompacted soil with Maris Piper (MPU) or Inca Bella (IBU), and compacted soil with Maris Piper (MPC) or Inca Bella (MPC). Each plot comprised a single ridge, with two rows of tubers planted 40 cm apart. Each ridge was 0.9 m in width. Each plot was ten tubers (3.6 m) long for a total of 20 tubers per plot.

Trial 2 took place on a loam soil (48% sand, 45% silt and 7% clay), with a previous crop of winter barley located at a latitude of 53.181980, and a longitude of -0.276750. The plots were irrigated with drip tape placed 5 cm below the level of the topsoil designed to apply 3.5 mm/day irrigation. Rainfall and temperature data was logged by an iMETOS 3.3 weather station (Pessl Instruments, Weiz, Austria). The field received 32 mm of rainfall in May after planting, 37 mm in June, 17 mm in July, 70 mm in August, and 25 mm in September before harvesting. Further weather data can be found in Data Sheet 2. The field was fertilized with 180 kg/ha nitrogen prior to planting. Differences in soil texture and nutrient content to trial 1’s site necessitated a different fertilization regime, following agronomic recommendations. Fourteen days prior to planting, the trial area was ploughed to a depth of 30 cm before bed forming. The trial was split into four plots on adjacent ridges with the same tuber spacing and treatments used in trial 1. Each plot was nine tubers (3.2 m) long, with a total of 18 tubers per plot.

Both trials had guard plots of the cultivar Taurus, and applied herbicide, blight spray and pre-harvest flailing during the growing season. All were applied by machinery at least 3 m outside the trial area to avoid compaction due to traffic.

### 2.3 Trial 1

The compacted treatment was produced by applying a 0.61 kN/m^2^ force by walking across the entire plot area twice, and soil resistance measured again. Soil resistance was measured immediately before planting using a hand penetrometer (Van Walt, Hazelmere, UK). Resistance was measured at 5 cm intervals either to a depth of 100 cm, or until resistance exceeded the penetrometer’s maximum value of 5 MPa. A soil moisture access tube (suitable for the PR2/4 profile probe, Delta-T Devices, Cambridge, UK) was inserted into each plot, 60 cm from the plot’s edge along the center line to allow soil moisture to be measured at 10 cm intervals to a depth of 40 cm.

After emergence, data was collected fortnightly. Soil moisture was measured using the profile probe. One shoot per plot was excised each visit to measure leaf area with a leaf area meter (Model LI-3100C, LI-COR, Nebraska, USA). One soil core (2.5 cm diameter, 15 cm depth, with three cores taken per location to a depth of 45 cm. Each core had a volume of 73.6 cm^3^) per plot was removed on each visit, adjacent to the excised plant stem. Soil tended to be too resistant to core beyond a depth of 45 cm. Root density was measured to compare the amount of root in each plot to changes in resistance. Root volume was measured using WinRHIZO software (Regent Instruments, Quebec, Canada) and used to calculate root density thus:


$$Root\;density\;\left(cm^{3}/cm^{3}\right)=\frac{Root\;Volume\;\left(cm^{3}\right)}{Soil\;Core\;Volume\;\left(cm^{3}\right)}$$

Soil resistance was measured immediately before harvesting, which occurred 116 days post-emergence. All tubers were manually harvested by plot and weighed to calculate yield.

### 2.4 Trial 2

The compacted treatment was produced by applying a 4.25 kN/m^2^ force to the topsoil by driving an Audi A4 All road vehicle across the whole plot area three times. Soil resistance was measured as in trial 1. As the soil was less resistant at depth, longer access tubes (suitable for the PR2/6 profile probe, Delta-T Devices, Cambridge, UK) were placed into each plot 80 cm from the plot edge along its length to allow soil moisture to be measured at 10, 20, 30, 40, 60 and 100 cm depth.

Soil moisture, root density and leaf area data were obtained as described for trial 1. Soil resistance was also measured fortnightly in this trial, as well as immediately before harvesting, which occurred 120 days post-emergence. All tubers were manually harvested by plot and weighed to calculate yield.

### 2.5 Statistical analyses

Statistical analysis was performed using Version 0.16 of JASP (University of Amsterdam, The Netherlands), with statistical significance ascribed when the p-value was less than 0.05. The controlled environment experiment utilized 2-way ANOVA with compaction and cultivar as independent variables, and leaf area and root density as dependent variables. Each field trial utilized 2-way ANOVA with compaction and cultivar as independent variables, and leaf area, root density, initial and final soil resistance, soil water content, and yield as dependent variables. These tests were also performed for soil water content and root density data from the last visit before harvest for each trial, as well as leaf area data from each measurement date and yield. With the same variables, 3-way ANOVA was utilized but also including trial as an independent variable to detect any variation between trials. Least square differences were calculated for soil resistance at each measured depth for both trials for each set of measurement. The relationship between soil resistance and moisture for the two trials was determined by using a linear regression with soil moisture as a dependent variable, and soil resistance and trial as independent variables. For each trial, variation in soil water content was determined using a linear regression with soil resistance, variety, and soil depth as covariates. Pearson correlation coefficients were calculated for each treatment from both trials with regards to initial and end soil resistance, soil moisture, leaf area, root density and yield. Linear regressions were used to determine the significance of these correlations.

## 3 Results

### 3.1 Controlled environment experiment

**Figure 1:**
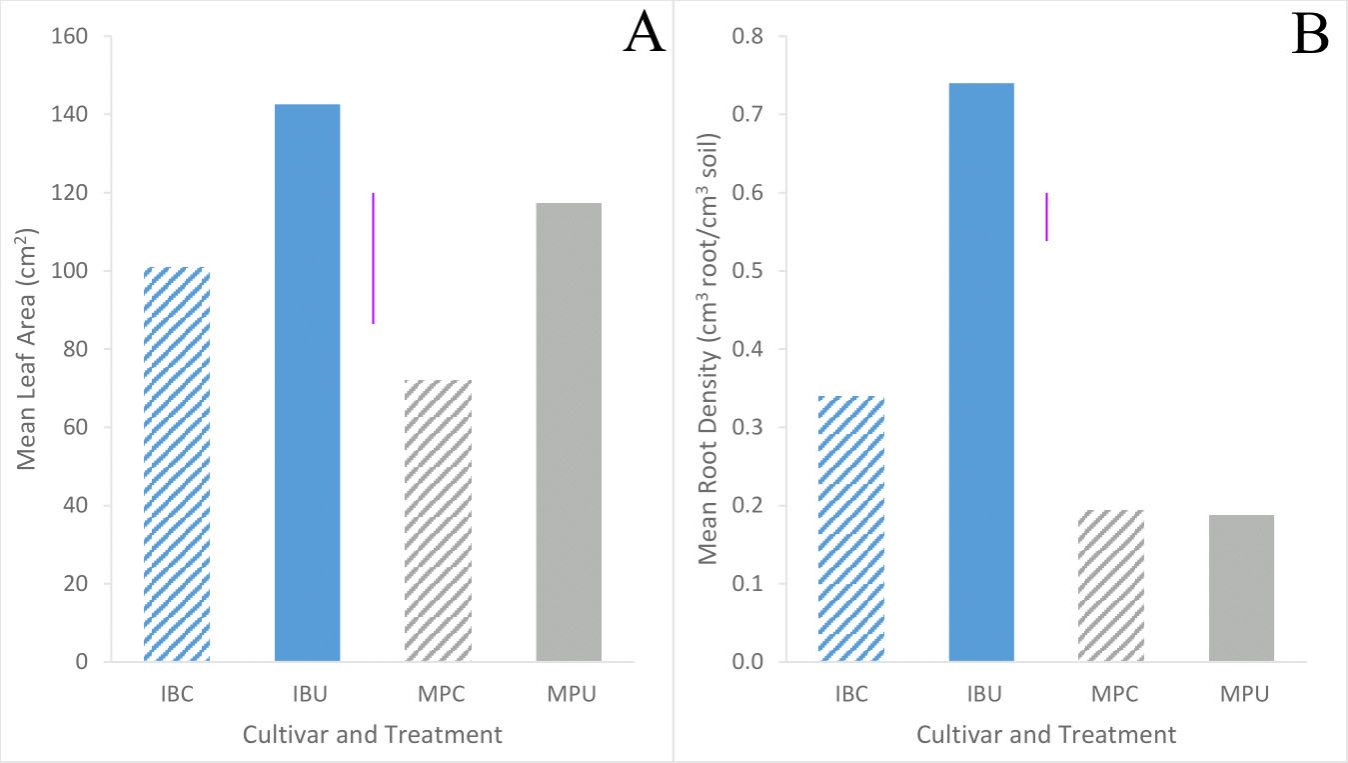
Mean leaf area **(A)** and root density **(B)** of Inca Bella (blue) and Maris Piper (grey) compacted (diagonal lines) pots or cultivars grown in loose (solid bar). Each bar represents five plants. LSD (5%) is given for each variable (purple lines).

Inca Bella tended to (p=0.07) produce greater leaf area, and a significantly larger root density (p< 0.001) than Maris Piper (**Table 1**). Soil compaction decreased leaf area in both cultivars (p = 0.001). Leaf area of Maris Piper decreased by 40%, whilst Inca Bella leaf area decreased by 30% (**Figure 1A**). Maris Piper plants produced a root system 40% the size of Inca Bella plants. Compaction-induced changes in root density substantially differed (p< 0.001) between cultivars. Root density of Maris Piper did not change (p = 0.82), but compacted soil decreased root density of Inca Bella by 65% (**Figure 1B**). Thus, shoot growth exhibited conserved responses to compaction, whilst root growth responses were cultivar-specific.

**Table 1:** Two-way analysis of variance (ANOVA) of cultivar and compaction on leaf area and root density in the controlled environment experiment.

Source of Variation	Leaf Area	Root Density
*Cultivar*	0.06	**<0.001**
*Compaction*	**0.001**	**<0.001**
*Cultivar x Compaction*	0.90	**<0.001**

Significant (P<0.05) p-values in bold text.

### 3.2 Soil measurements

**Figure 2:**
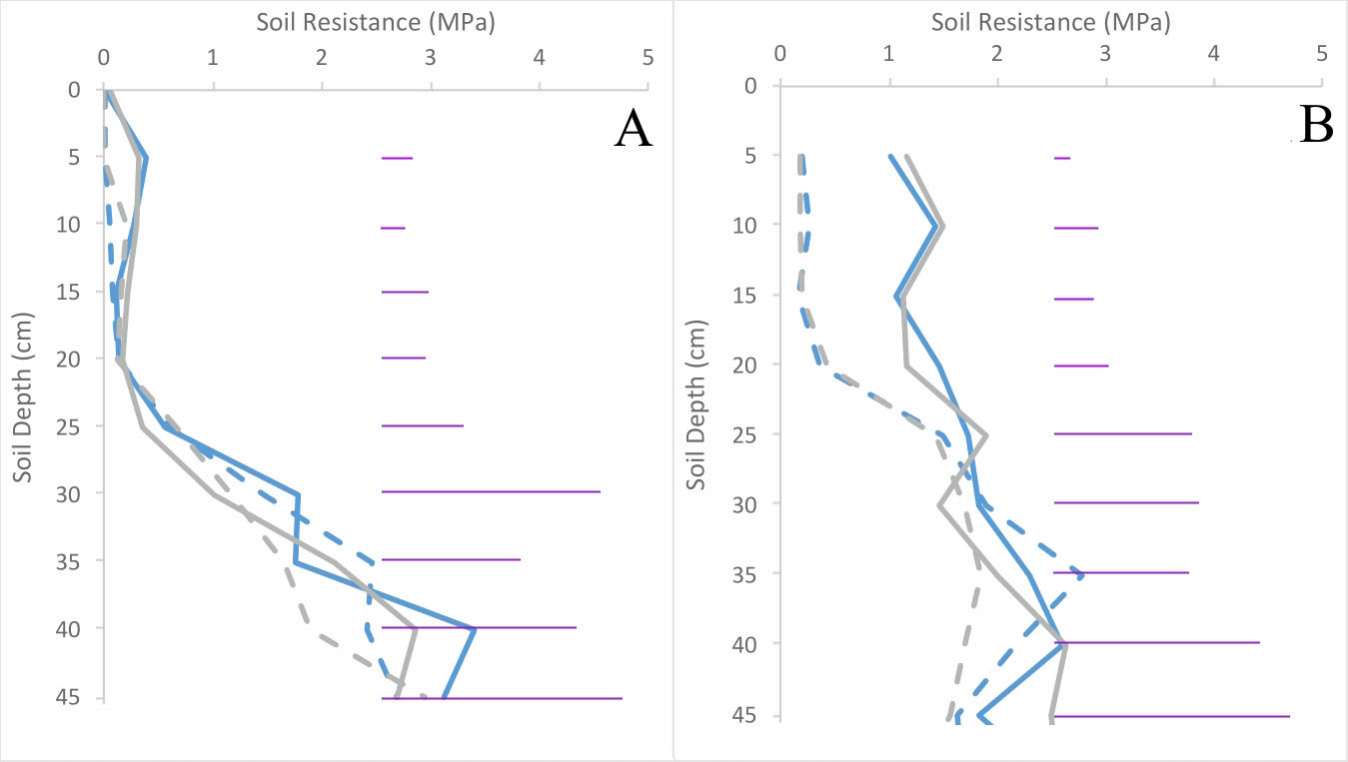
Soil resistance immediately after compaction was applied in trials 1 **(A)** and 2 **(B)** for both Inca Bella (blue) and Maris Piper (grey) plots. Compacted and uncompacted plots are indicated with unbroken lines and dashed lines respectively. Each point represents three resistance measurements. LSD (5%) is given for each depth (purple horizontal lines).

Soil resistance in compacted plots was three-fold higher (p< 0.001) in trial 2 than trial 1 after planting, with no variation in resistance between cultivars at this stage (p = 0.67) (**Figure 2**). Compacting the soil doubled resistance in the upper 20 cm of soil in trial 1, and increased it five-fold in trial 2 (**Figure 2**). No significant differences were observed between treatments below 10 cm in trial 1, and below 20 cm in trial 2.

**Figure 3:**
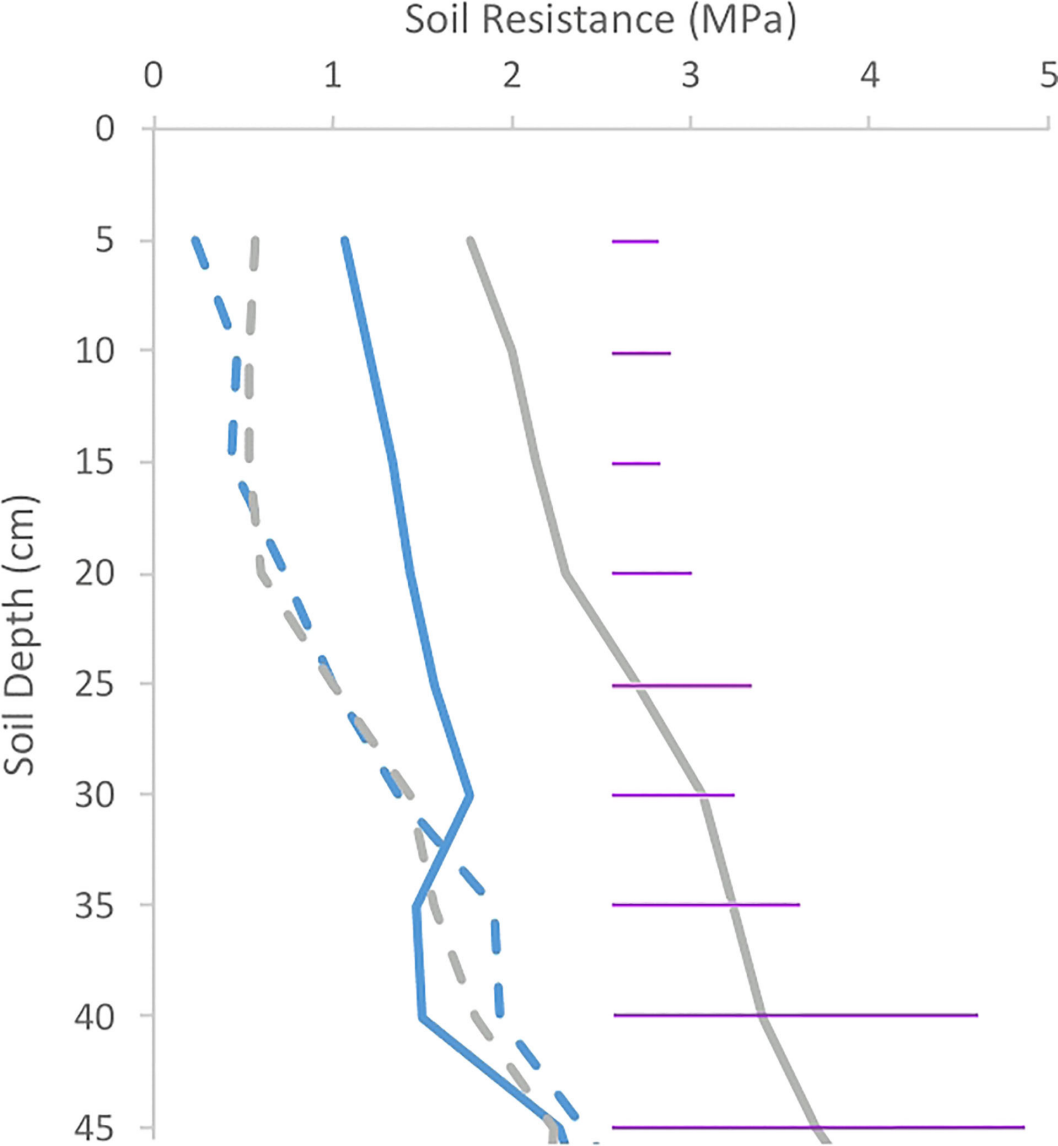
Soil resistance of plots in trial 2 49 days after emergence in trial 2 for both Inca Bella (blue), and Maris Piper (grey). Compacted and uncompacted plots are indicated with unbroken and dashed lines respectively. Each point represents three measurements. LSD (5%) is given for each depth (purple horizontal lines).

When soil resistance was measured 49 days after emergence in trial 2, resistance of Inca Bella plots had increased 1.2-fold since planting, but resistance of the Maris Piper plots had increased 1.7-fold (**Figure 3**). Resistance in the uncompacted plots tended to slightly increase (p = 0.08). In the compacted plots, resistances significantly differed in the upper 35 cm between the two cultivars until harvest.

**Figure 4:**
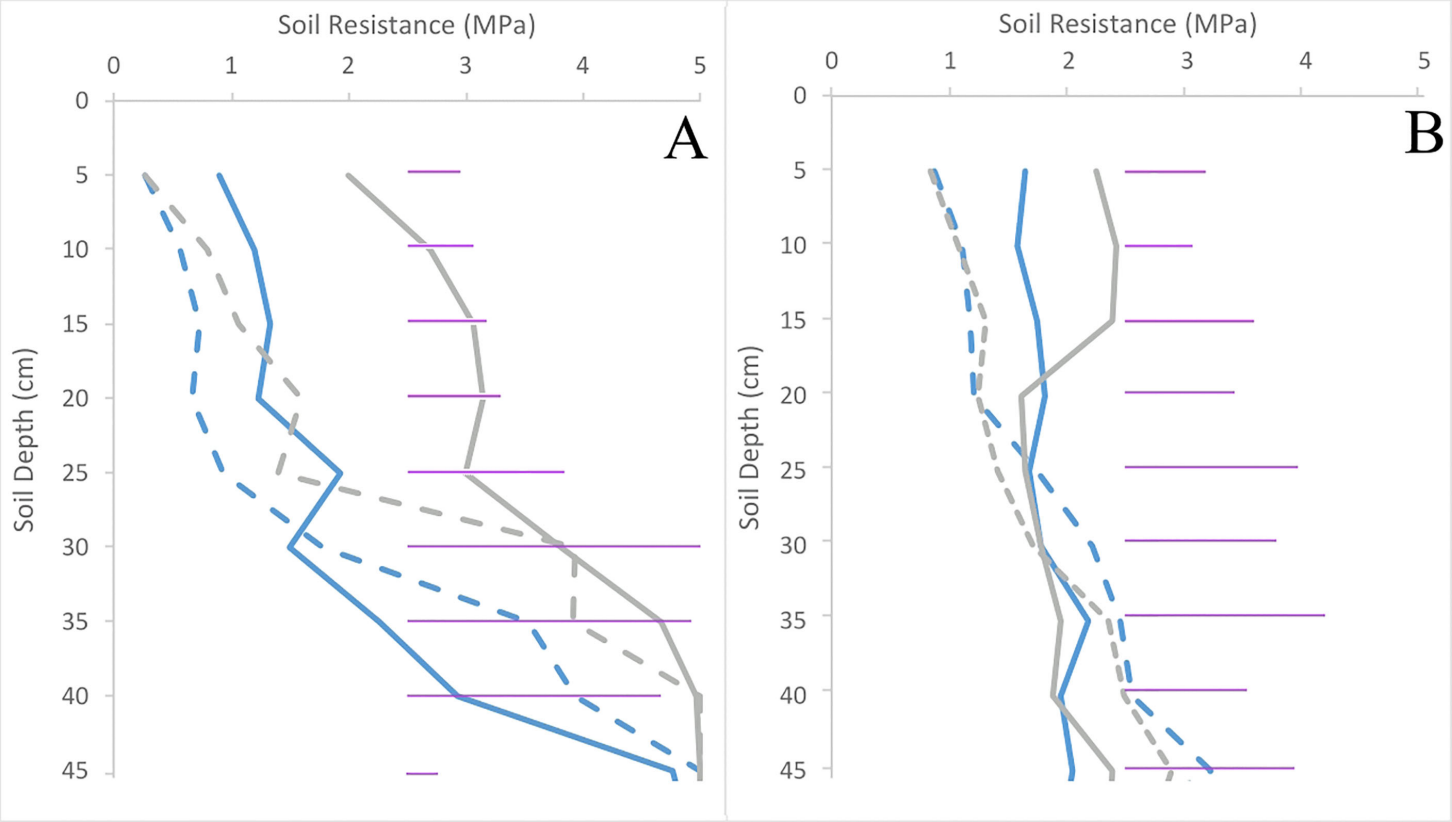
Soil resistances of plots in trials 1 **(A)** and 2 **(B)** for both Inca Bella (blue) and Maris Piper (grey) at harvest. Compacted and uncompacted plots are indicated with unbroken and dashed lines respectively. Each point represents three resistance measurements. LSD (5%) is given for each depth (purple horizontal lines).

Across the entire soil profile in both trials, there were very clear differences in soil resistance within the upper 15 cm of the soil (p< 0.001). Despite the 7-fold increase in initial compacting pressure, soil resistance at harvest time was similar (p = 0.43) between trials. Compared to uncompacted plots, soil resistance of the compacted plots increased 2.5-fold in Inca Bella and 3-fold in Maris Piper in trial 2 (**Figure 4**).

**Figure 5:**
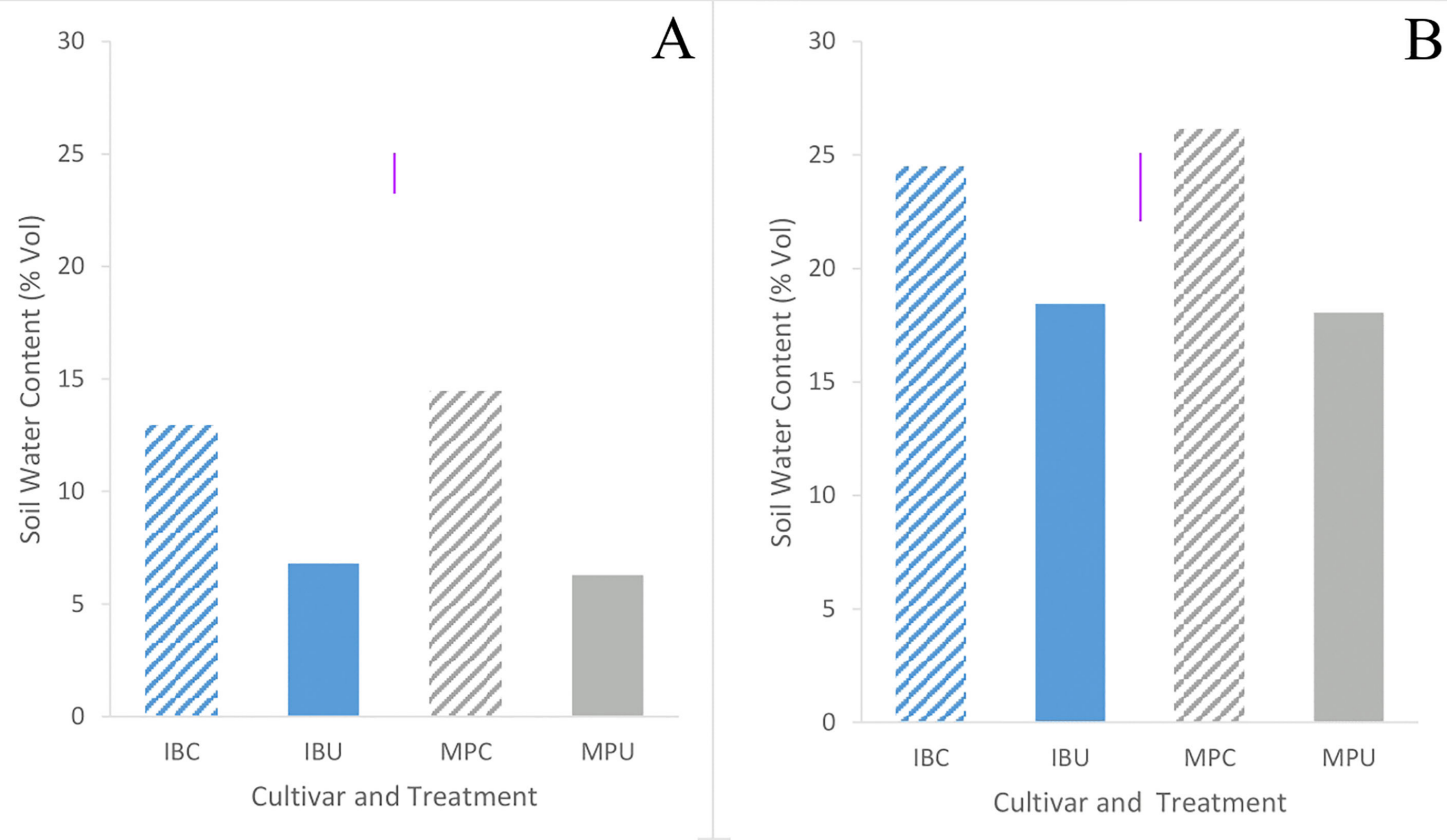
Mean soil water content values in the upper 20 cm of the soil two weeks before harvest in trials 1 **(A)** and 2 **(B)** of Inca Bella (blue) and Maris Piper (grey) cultivars grown in compacted (diagonal lines) or uncompacted (solid bars) soil. Each bar represents 6 data points, 3 from 10 cm soil depth and 3 from 20 cm. Soil moisture within each plot did not differ between these depths. LSD (5%) is given for each trial.

Soil water content was 60% lower in trial 1 than trial 2 across all treatments. Soil water content of uncompacted plots averaged 50% less (p< 0.001) than compacted plots in trial 1, and 30% in trial 2 (**Figure 5**). Soil water content did not differ (p = 0.75) between cultivars in either trial (**Table 2**). Soil water content data for all depths across the growing season in trials 1 and 2 can be found in **Supplementary Figures 1** and **2** respectively.

**Table 2:** Two-way ANOVA of cultivar, compaction, and their interaction on soil resistance in the upper 20 cm of the soil at the start and end of the growing season, along with soil water content, leaf area and root density at the end of the growing season, and yield in the two trials.

Source of Variation	Initial Resistance		End Resistance		Soil Water Content		Leaf Area		Root Density		Yield	
	Trial 1	Trial 2	Trial 1	Trial 2	Trial 1	Trial 2	Trial 1	Trial 2	Trial 1	Trial 2	Trial 1	Trial 2
*Cultivar (cv)*	0.26	0.98	**<0.001**	**0.04**	0.72	0.95	0.37	**0.008**	0.28	0.62	**<0.001**	0.09
*Compaction (co)*	**<0.001**	**<0.001**	**<0.001**	**<0.001**	**<0.001**	**0.02**	0.65	0.77	0.31	0.79	0.26	**0.03**
*cv x co*	0.66	0.98	**<0.01**	**0.03**	0.47	0.59	0.32	0.37	0.12	0.68	0.23	0.88

Significant (P<0.05) p-values in bold text.

**Figure 6:**
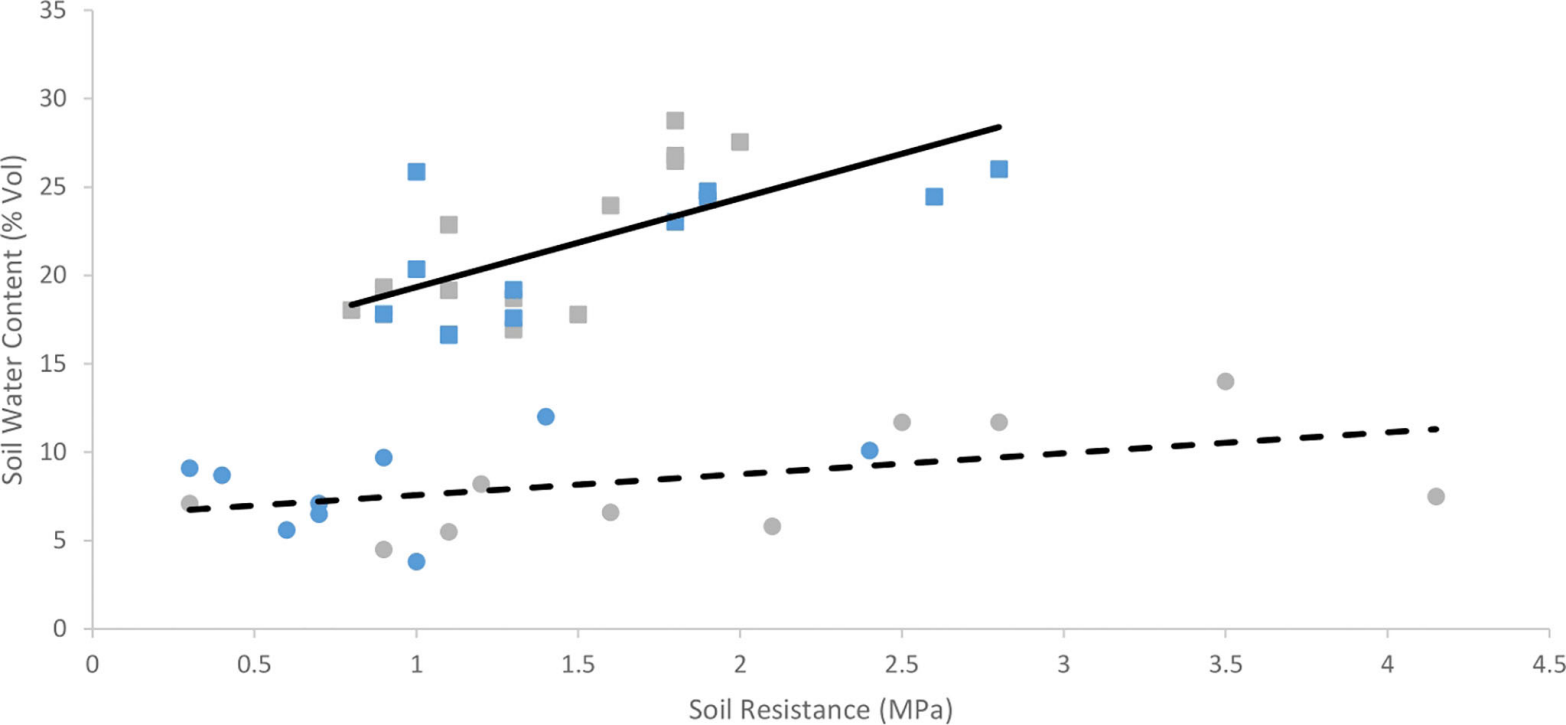
The relationship between soil moisture and soil resistance in the upper 20 cm of the topsoil two weeks before harvest. Each point represents paired data of resistance from an individual Inca Bella (blue) or Maris Piper (grey) plot from across each growing season. Trial 1 (circles) had the linear regression equation *y* = 1.18*x* + 6.39 and an R^2^ value of 0.22, shown as the dashed line. Trial 2 (squares) had the linear regression equation *y* = 5.03*x* + 14.31 and an R^2^ value of 0.44, shown as the solid line.

In measurements taken two weeks before harvest, soil resistance increased with soil moisture in both trials (p< 0.001). This relationship varied between trials (**Figure 6**), with soil moisture affecting soil resistance less in trial 1.

### 3.3 Plant measurements

**Figure 7:**
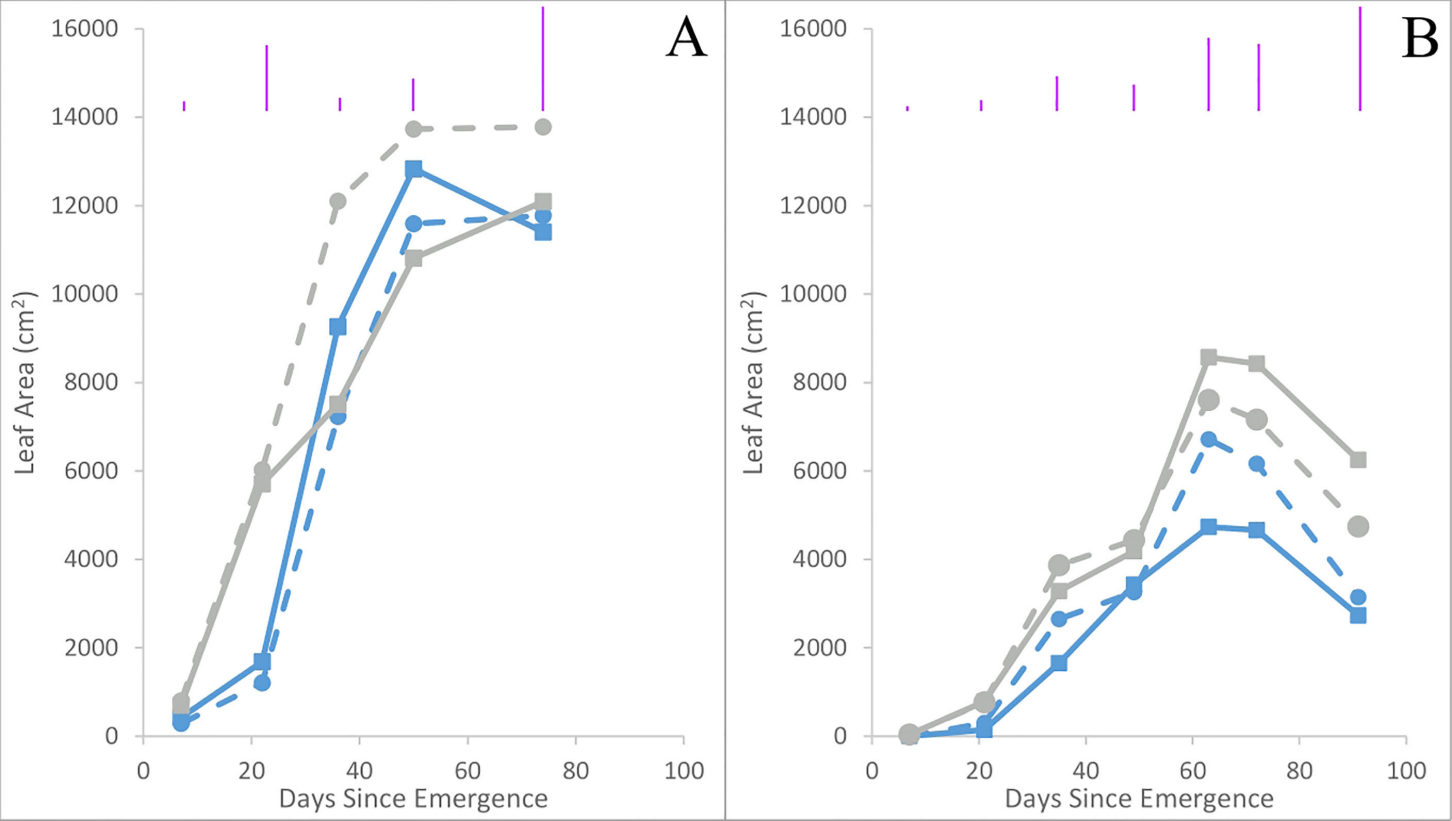
Mean leaf area in trials 1 **(A)** and 2 **(B)** for Maris Piper in compacted soil (grey squares, solid line) and uncompacted soil (grey circles, dashed line), and Inca Bella plants grown in compacted soil (blue squares, solid line) or uncompacted soil (blue circles, dashed line). Each point represents a mean of three plants. LSD (5%) is given for each measurement date (purple lines). Significances can be found in **Table 4**.

**Figure 8:**
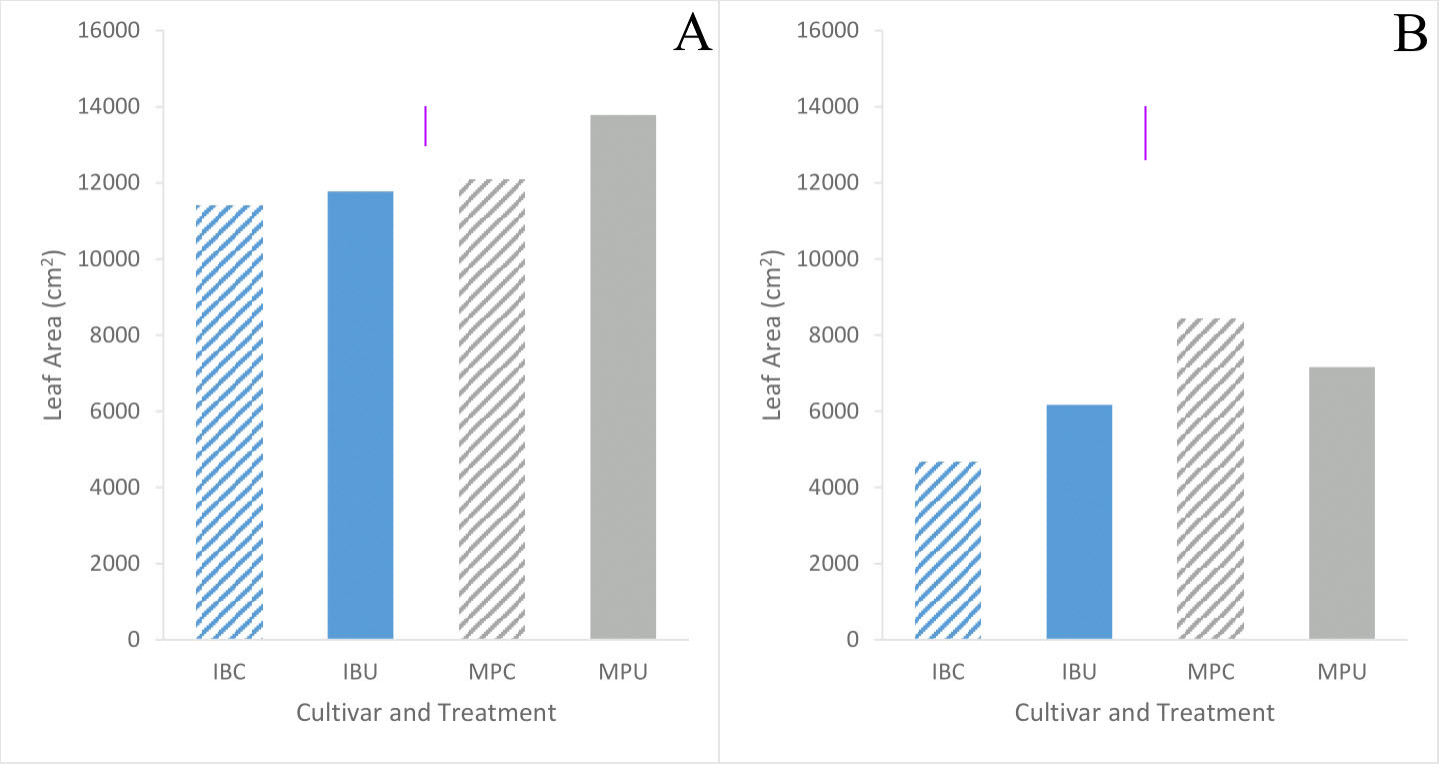
Mean leaf area of each treatment 72 days after emergence in trial 1 **(A)**, and 74 days after emergence in trial 2 **(B)** of Inca Bella (blue) and Maris Piper (grey) cultivars grown in compacted (diagonal lines) or uncompacted (solid bar) soil. LSD (5%) is given for each trial (purple lines).

Leaf area tended to be higher in Maris Piper than Inca Bella (p = 0.06) across both trials (**Table 3**). Averaged across all treatments and measurement occasions, leaf area was 70% greater in trial 1 than trial 2. Leaf area of Inca Bella was substantially less than Maris Piper when measured up to 49 days after emergence in trial 1 (**Figure 7**), and on all sampling dates except 49 days after emergence in trial 2 (**Table 4**). Near the time of maximum canopy development in trial 1, Maris Piper plants grown in uncompacted plots had 15% larger leaf area than those in compacted soil, while soil compaction had no effect on Inca Bella leaf area (**Figure 8A**). At a similar phenological stage in trial 2, Maris Piper plants in compacted plots had 15% larger leaf area than those in uncompacted plots, but Inca Bella plants grown in compacted soil had 25% less leaf area than those in uncompacted soil. Overall, leaf area of Maris Piper tended to be more responsive to soil compaction than Inca Bella.

**Table 3:** Three-way ANOVA of cultivar, compaction, and trial on soil resistance in the upper 20 cm of the soil at the start and end of the growing season, along with soil water content, leaf area and root density at the end of the growing season, and yield.

Source of Variation	Initial Resistance	End Resistance	Soil Water Content	Leaf Area	Root Density	Yield
*Trial (t)*	**<0.001**	0.43	**<0.001**	**<0.001**	0.64	**0.007**
*Cultivar (cv)*	0.66	**<0.001**	0.82	0.06	0.77	**<0.001**
*Compaction (co)*	**<0.001**	**<0.001**	**0.003**	0.59	0.51	**0.012**
*t x cv*	0.63	**<0.001**	0.99	0.76	0.33	**0.001**
*t x co*	**<0.001**	**0.044**	0.94	0.77	0.99	0.16
*cv x co*	0.84	0.16	0.57	0.59	0.30	0.40
*t x cv x co*	0.88	**<0.001**	0.91	0.20	0.99	0.55

Significant (p< 0.05) p-values are written in bold text.

**Table 4:** Two-way ANOVA table of cultivar and compaction on leaf area at each measurement date across both trials.

Source of Variation	7 Days Since Emergence		21 Days Since Emergence		35 Days Since Emergence		49 Days Since Emergence		63 Days Since Emergence	72 Days Since Emergence		91 Days Since Emergence
	T1	T2	T1	T2	T1	T2	T1	T2	T2	T1	T2	T2
*Cultivar (cv)*	**<0.001**	**0.04**	**<0.001**	**<0.001**	**<0.001**	**<0.001**	0.83	**0.03**	**<0.01**	**<0.01**	**<0.01**	**<0.01**
*Compaction (co)*	0.82	0.83	0.91	0.51	**<0.001**	0.24	**0.01**	**0.04**	0.37	**0.02**	0.29	0.46
*co x cv*	0.17	0.92	0.58	0.41	**<0.001**	**0.04**	**<0.001**	**0.01**	**0.02**	0.31	**0.05**	0.20

Significant (p< 0.05) p-values are written in bold text.

**Figure 9:**
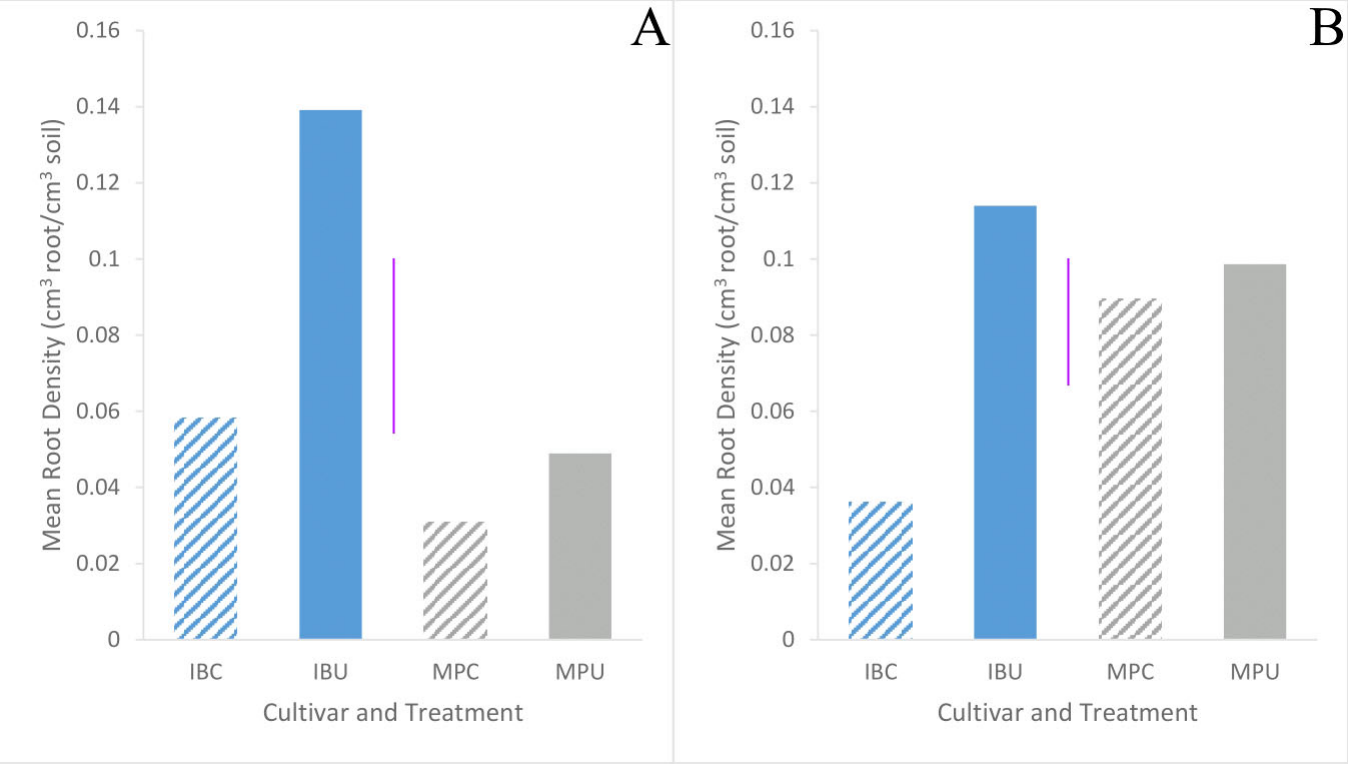
Mean root density of Inca Bella (blue) and Maris Piper (grey) grown in compacted (diagonal lines) and uncompacted (solid bars) soil from soil cores taken between 0 cm and 15 cm depth on the last field visit before harvest: 102 days after emergence in trial 1 **(A)** and 89 days after emergence in trial 2 **(B)**. Each bar represents six replicates. LSD (5%) is given for each trial (purple lines).

Root density did not differ (p = 0.77) between cultivars (**Table 3**). At harvest, Inca Bella root density did not significantly differ between trials, but Maris Piper root density in trial 2 was 7-fold higher in the compacted treatment and 3-fold higher in the uncompacted treatment than in trial 1 (**Figure 9**). Inca Bella root density was similar in the upper 15 cm of soil between both trials. Root density with depth was unaffected by soil compaction. Maris Piper maintained a similar root density in both compacted soil and uncompacted soil in both trials, whilst Inca Bella root density tended to decrease in compacted soil (**Figure 9**). Overall, root density of Inca Bella was more sensitive to compaction than Maris Piper.

**Figure 10:**
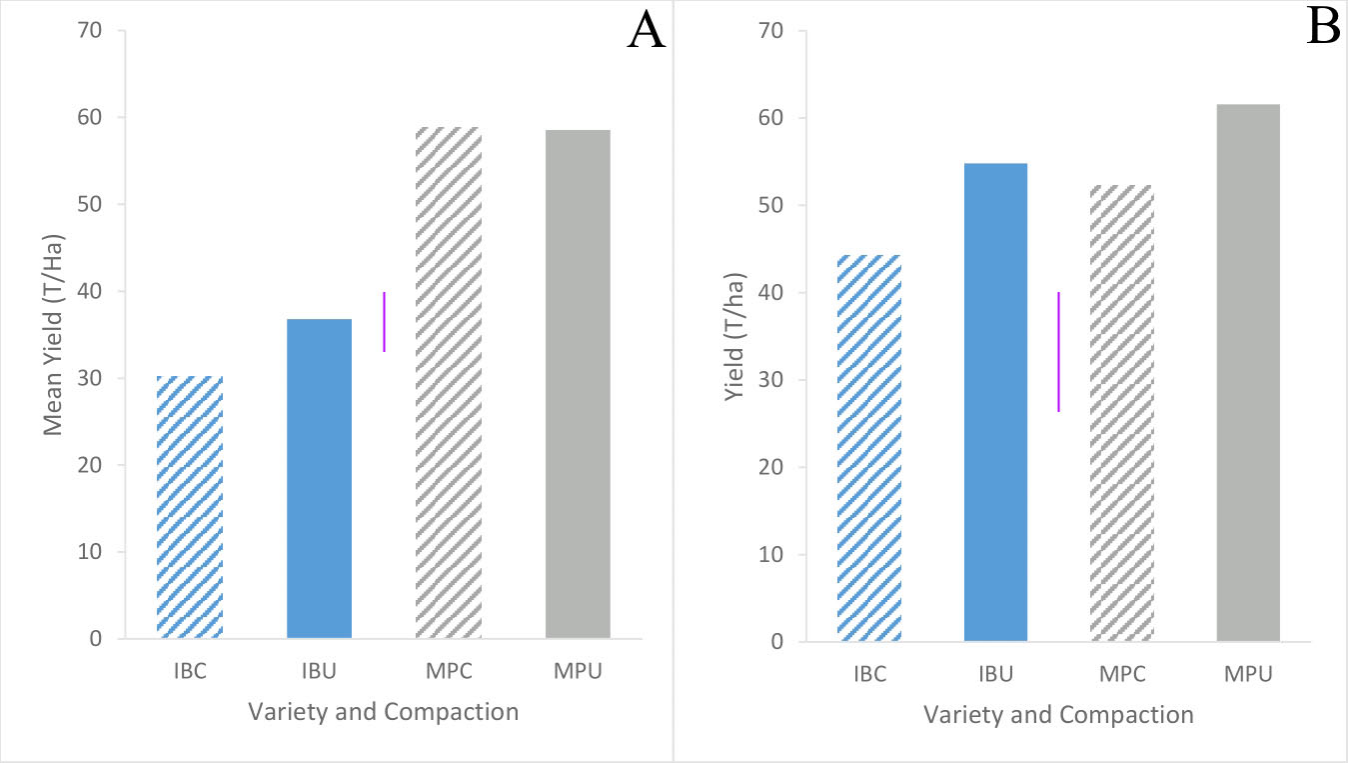
Mean gross yield of Inca Bella (blue) and Maris Piper (grey) grown in compacted (diagonal lines) and uncompacted (solid bars) in trials 1 **(A)** and 2 **(B)**. Each bar represents the mean of three plots. LSD (5%) is given for each trial (purple lines).

Although Maris Piper yield did not differ between trials (p = 0.67) and averaged 57.8 t/ha, Inca Bella yield was greater in trial 2 (49 t/ha) than trial 1 (33 t/ha) (**Figure 10**). Compacted soil decreased mean tuber yield by 20% in trial 2 but had less effect in trial 1 (**Table 2**). However, soil compaction decreased Inca Bella yield by a mean 20% (p = 0.01). Maris Piper yield was not affected by soil compaction (p = 0.27). Overall, yield of Inca Bella was more affected by soil compaction than yield of Maris Piper.

Across both cultivars and treatments, yield was not significantly correlated with any of the measured variables, although leaf area explained more of the variation in yield (R^2 = ^0.56) than any other (**Table 5**). Soil water content on the last field visit was positively correlated with initial soil resistance, and negatively correlated with maximum leaf area (**Table 5**).

**Table 5:** Pearson correlation coefficients for variables across all plots from both trials.

Initial Resistance	1.00					
End Resistance	0.33	1.00				
Soil Water Content	0.80**	0.50	1.00			
Leaf Area	-0.58	-0.02	-0.82*	1.00		
Root Density	0.34	-0.30	0.43	-0.54	1.00	
Yield	-0.31	-0.08	-0.40	0.56	0.21	1.00
	Initial Resistance	End Resistance	Soil Water Content	Leaf Area	Root Density	Yield

Purple coloured boxes indicate positive correlations, and red boxes indicating negative correlations. Stronger correlations are darker coloured. Significant correlations in bold text with asterisks indicating level of significance. * = p< 0.05, ** = p< 0.01.

## 4 Discussion

### 4.1 Effects of soil resistance on potato plants

Whereas Maris Piper root growth was unaffected by soil compaction in both controlled environment (**Figure 1B**) and field experiments (**Figure 9**), Inca Bella was more sensitive to increased soil compaction with 50% less root growth in both environments. Although rooting density was typically lower in the field trial, likely due to increased growing space, cultivar sensitivity to soil compaction was conserved. Whilst potato rooting traits in the field were comparable to those observed in controlled environment experiments by using sufficiently large pots to avoid root restriction (Wishart et al., 2013), responses to stresses such as drought (Puértolas et al., 2014) are less consistent between field trials and controlled environment experiments. This occurs as drought stressed plants grow deeper roots to extract water from deeper soil layers (Pierret et al., 2016) not existing in pots. However, soil resistance below 35 to 45 cm restricted root growth (Stalham et al., 2007) in trial 1 (**Figure 2**), which limited root growth to the topsoil where soil resistance tended to be consistent with depth (**Figure 4**), as in the controlled environment experiment. When root-inhibiting compaction at depth prevents deep rooting, such as in trial 1 (**Figure 4**), these results support the hypothesis that controlled environment experiments can help predict varietal differences in root growth responses to compaction in field soils (Colombi and Walter, 2016). Reduced root density in the compacted treatment can be attributed to either increased soil water content, or increased soil resistance. Despite the higher soil water content in trial 2 (**Figure 5**), root density did not significantly differ between trials (**Table 3**). Higher soil water content in compacted soil (**Figure 5**) could reduce aeration (Ghosh and Daigh, 2020) and soil oxygen availability and thus root growth (Nguyen et al., 2020). As soil compaction decreased Inca Bella root density similarly in both trials (**Figure 9**), despite higher soil water content in trial 2, it is unlikely that poor soil aeration is responsible for the lower root density in compacted soil.

Soil resistance in the upper 20 cm of soil increased in the compacted treatment over time (**Figures 2**–**4**). Potato roots continue to grow until two to three weeks before harvest (Iwama, 2008), with increased soil resistance in the compacted treatment since tuber initiation (**Figure 3**) inhibiting root growth (**Table 5**), leading to reduced root density (Huntenburg et al., 2021). Potato root systems are shallower and less dense than most crop plants (Yamaguchi and Tanaka, 1990; Iwama, 2008). Proportionally more roots occur in the upper, compacted soil layers, with more than 50% of roots concentrated in the upper 30 cm (Stalham et al., 2007). Together, these factors result in much of the root system growing through inhibitory levels of soil resistance. Soil resistances greater than 1 MPa limit root growth of Maris Piper, and inhibit it completely at values over 3 MPa (Stalham et al., 2007). Whilst initial resistance values in trial 1 were below these thresholds for both treatments, and the compacted treatment in trial 2 was barely sufficient to restrict root growth (**Figure 2**), soil resistance was sufficiently high in all compacted treatments to restrict root growth by the time of harvest (**Figure 4**). While Inca Bella root density was greatly reduced in the compacted soil, Maris Piper root growth was more tolerant (**Figure 9**), which indicates genotypic differences in the ability to tolerate increased soil resistance.

Leaf area was consistently larger for all plants in trial 1 than trial 2 (**Figure 7**). This may be due to higher rainfall throughout the growing season of trial 1 (Data Sheet 1; Data Sheet 2). Maximum leaf area tended to be similar for both cultivars in trial 1 despite limited early growth of Inca Bella, while Maris Piper produced a larger canopy in trial 2 (**Figure 7**). In contrast, Maris Piper tended to produce a smaller canopy than Inca Bella in the controlled environment trial. Whilst compaction inhibited early canopy growth, there was no overall effect of compaction on final leaf area. Shoot growth has often been correlated with potato yield, with leaf area index (R^2 = ^0.77 – Luo et al., 2020) and mid-season biomass (R^2 = ^0.71 – Huntenburg et al., 2021) being accurate predictors. In these trials, maximum leaf area and yield were less strongly correlated (R^2 = ^0.56), but leaf area explained more of the variation in yield than other measured variables (**Table 5**). *Phureja* group cultivars typically produce slower-growing canopies than *tuberosum* cultivars, which are more resistant to reduced water availability (Wishart et al., 2014). The lack of irrigation in trial 1 may therefore explain why maximum leaf area was similar between cultivars in trial 1, despite Inca Bella producing a larger canopy than Maris Piper in the controlled environment experiment (**Figure 1**). It is also possible that Inca Bella plants are more vulnerable to stresses other than compaction that were not present in the controlled environment experiment, such as heat stress. Ambient temperatures reached almost 40°C during trial 2’s growing season, and some evidence suggests that *phureja* group potatoes are less tolerant of heat stress than those from the *tuberosum* group (Hetherington et al., 1983). This, combined with soil compaction stress may have limited Inca Bella early canopy growth more than in Maris Piper.

Although compacted soil had variable impacts on Inca Bella leaf area (no effect in trial 1, 25% reduction in trial 2), yield declined by a mean 20% across both trials (**Figure 10**). Yields of Maris Piper seemed unaffected by compaction, with plants in compacted soil tending to have a greater leaf area than those in uncompacted plots in trial 2, but the opposite response in trial 1 (**Figure 8**). Increased canopy area improves radiation interception and thus carbon fixation (Huntenburg et al., 2021), but leaf growth tends to reach its maximum around fifty to sixty days after emergence (**Figure 7**). At this time, cultivar-dependent differences in soil resistance begin to occur, so the limited correlation between yield and maximum leaf area is likely due to soil resistance increasing after canopy closure, affecting yield without affecting initial canopy growth.

While soil resistance of agricultural fields can increase throughout a growing season (Whalley et al., 2006), especially when compaction is applied (Huntenburg et al., 2021), cultivar-dependent effects on soil resistance have not previously been observed. Soil resistance increased to similar, cultivar-dependent values in the compacted treatments (**Figure 4**), irrespective of the amount of initial compaction (**Figure 2**). As this increase was not observed in uncompacted plots, initially compacting the soil seems to be necessary for cultivar-dependent increases in soil resistance to arise.

### 4.2 Effect of potato plants on soil resistance

Soil water content was significantly higher in trial 2 than in trial 1 (**Figure 6**). This is likely caused by trial 1 being rainfed, whereas trial 2 was fully irrigated. In addition, silty loam soils typically hold more water than loam soils (Saxton et al., 1986). Although no physical signs of drought stress were observed, soil water content of uncompacted plots in trial 1 were estimated to be close to permanent wilting point (**Figure 5**). Soil water content was originally hypothesized to be lower in compacted soils, as these have reduced water holding capacity (Smith et al., 2001) due to reduced pore density (Batey, 2009). Furthermore, compacted soil loses water via evaporation up to 50% faster than uncompacted soil with the same soil moisture (An et al., 2018) as mean pore size is decreased, increasing capillary suction (Goldberg-Yehuda et al., 2022). However, compacted soil was wetter than uncompacted soil across both trials at time of harvest (**Figure 5**). Furthermore, the tendency towards reduced leaf area (**Table 4**) and root density (**Figure 9**) in compacted soil likely decreased crop water uptake and water demand (Stalham and Allen, 2004), thus contributing to greater soil moisture in the compacted plots. Although penetration resistance typically increases as a soil dries (Batey, 2009; Vaz et al., 2011), the opposite relationship occurred when comparing compaction treatments (**Figure 6**). Thus, increased soil resistance of the compacted plots from beginning to the end of the season cannot be explained by soil moisture depletion. Since root density accounted for at most 0.3% of soil volume, with larger root systems in uncompacted soils, it is unlikely to cause seasonal increases in soil resistance. Instead, uncompacted Inca Bella plots had the highest root density (**Figure 9**) and lowest soil resistance (**Figure 4**). Furthermore, increased root density in the bulk soil is hypothesized to mitigate increases in soil resistance from traffic effects (Forster et al., 2020). While increased root density can increase soil resistance in the immediate vicinity of the root (Lucas et al., 2019), it is unlikely to have had any measurable effect on bulk soil resistance.

Whilst changes in root density cannot account for increases in soil resistance, potato tuber expansion may be important. Multiplying tuber density (*circa* 1067 kg m^-3^ - Patel et al., 2018) by yield per plot (**Figure 10**) can determine the proportion of soil volume occupied by tubers. Tubers tend to be limited to the upper 20 cm of soil (Matteau et al., 2022), and soil volume to this depth ranged from 0.576 m^3^ (trial 2) to 0.648 m^3^ (trial 1) in each plot according to its dimensions. Thus, the proportion of tuber volume in the topsoil at harvest was estimated to range from 4.6% to 9.9%, which could easily account for an increase in soil resistance as tuber expansion compacts the soil around the tubers. Furthermore, significant treatment differences in penetration resistance in trial 2 first arose 49 days post-emergence (**Figure 3**) coinciding with the start of the tuber bulking phase; when tubers begin to grow from small buds on the stolons between 39 and 55 days after emergence (Alva, 2004). Together, these data strongly imply that tuber bulking is primarily responsible for the observed seasonal increases in soil resistance.

That tuber bulking itself can cause root growth-limiting soil compaction can also explain data obtained in other experiments. Attempts to remove soil compaction by subsoiling have frequently been unsuccessful in restoring yield, with less than half of subsoiling trials significantly increasing yield, and those that did tending to show small (less than 5 t/ha) increments (Stalham et al., 2005). In fields with a high susceptibility to compaction, tuber growth rapidly increases the soil’s resistance and may inhibit yield in sensitive cultivars. Possibly tuber bulking increases soil resistance to the point that it inhibits further tuber growth, which explain the cultivar-dependent final soil resistances observed in the compacted treatments of both trials (**Figure 4**). Increased Inca Bella yield in compacted plots in trial 2 compared to trial 1 (**Figure 10**) was correlated with increased soil moisture (**Figure 5**) reducing soil resistance, allowing increased tuber growth before the maximum is reached. The relative insensitivity of Maris Piper yields to soil compaction may be due to genotypic differences in tuber growth at higher soil resistances, which were insufficient to inhibit Maris Piper tuber growth but constrained Inca Bella tuber growth. Some initial compaction facilitated these cultivar-dependent differences in soil resistance, with rainfall furthering compacting the topsoil (Augeard et al., 2008), preventing tuber expansion increasing soil height. Furthermore, as dryer soils are less susceptible to compaction (Ahmadi and Ghuar, 2015), soil resistance in the dryer uncompacted plots changed less as tuber biomass increased (**Figures 4**, **5**).

## 5 Conclusions

Potato tuber bulking seems responsible for increasing soil resistance during the growing season. Maris Piper produced higher yield and increased soil resistance to a greater amount than Inca Bella, indicating genotypic difference in compaction tolerance. The ability to maintain yield in compacted soil was correlated more with the ability to maintain canopy growth than root growth, suggesting that canopy growth can help to predict yield in compacted soil. Surface soil compaction appears necessary for the cultivar-dependent changes in soil resistance to occur, and could potentially be applied to potato fields by farm traffic throughout the growing season. Future research should focus on preventing growth-inhibiting compaction from occurring during tuber bulking, with increased irrigation at this time to reduce soil resistance allowing tuber bulking to occur unobstructed. Choosing appropriate cultivars could help farmers adapt to soils that are susceptible to compaction.

## Data availability statement

The raw data supporting the conclusions of this article will be made available by the authors, without undue reservation.

## Author contributions

PS wrote the manuscript, with editorial assistance from ID and DN. Experiment methodology was produced PS with suggestions from ID and DN. Data collection and analysis was undertaken by PS. Fieldwork was carried out by PS with help planting and harvesting by DN. All authors contributed to the article and approved the submitted version.
