# Supplementary material for: Cultivar-dependent differences in tuber growth cause increased soil resistance in potato fields
Source: Front Plant Sci. 2023 Jun 5;14:1095790. doi: 10.3389/fpls.2023.1095790 (PMC10278232; doi:10.3389/fpls.2023.1095790)
Supplement: Supplementary file 3 [file DataSheet_3.docx]

# Supplementary Figures


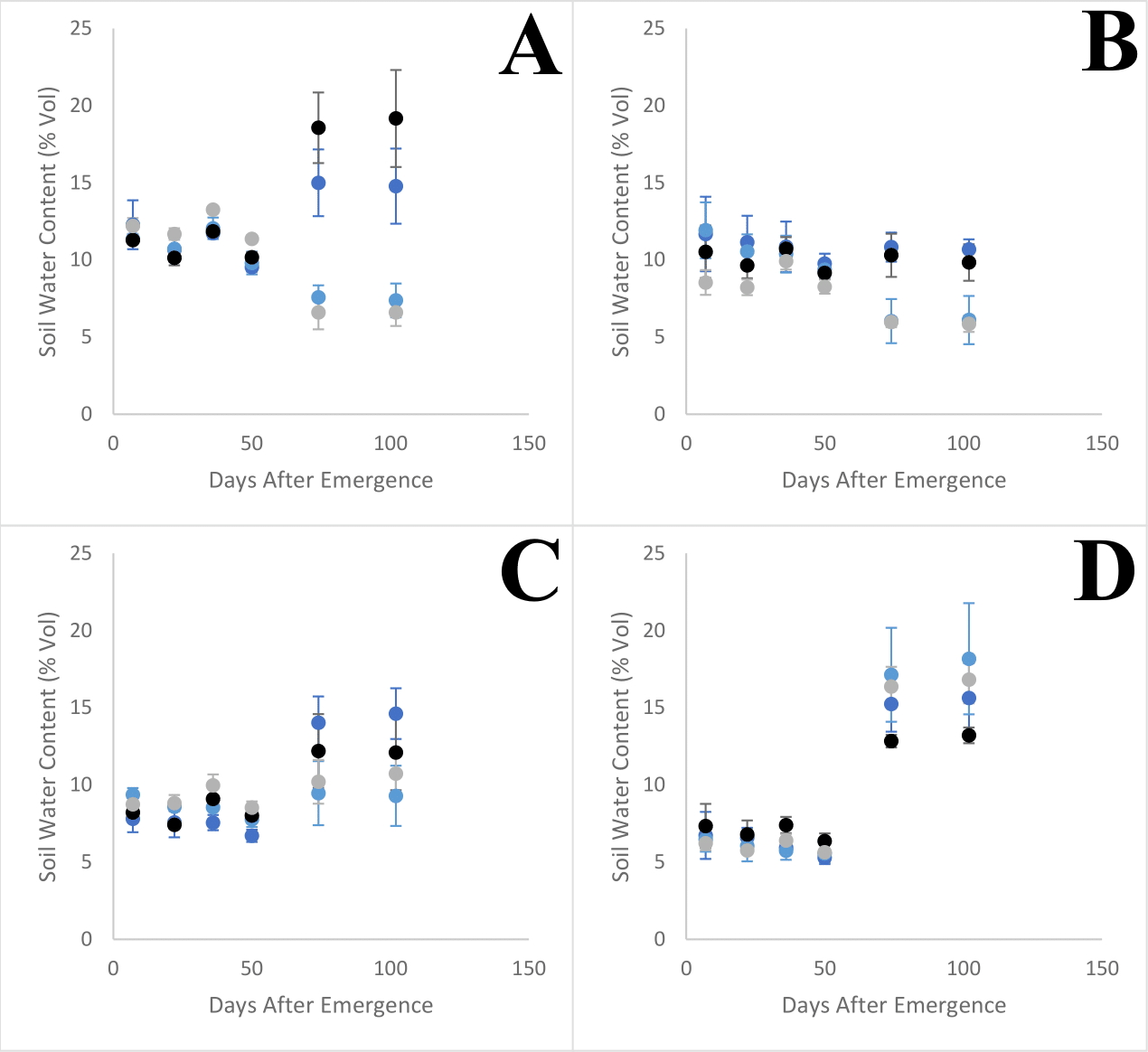


**Supplementary Figure 1.** Changes in soil water content over time in trial 1 at depths of 10 cm (A), 20 cm (B), 30 cm (C) and 40 cm (D). Each point represents the mean of three measurements of soil water content from either Inca Bella (blue) or Maris Piper (black/gray) plot with compacted (squares) or uncompacted (circles) treatment applied. Error bars indicate standard error with a p-value of 0.05.

**
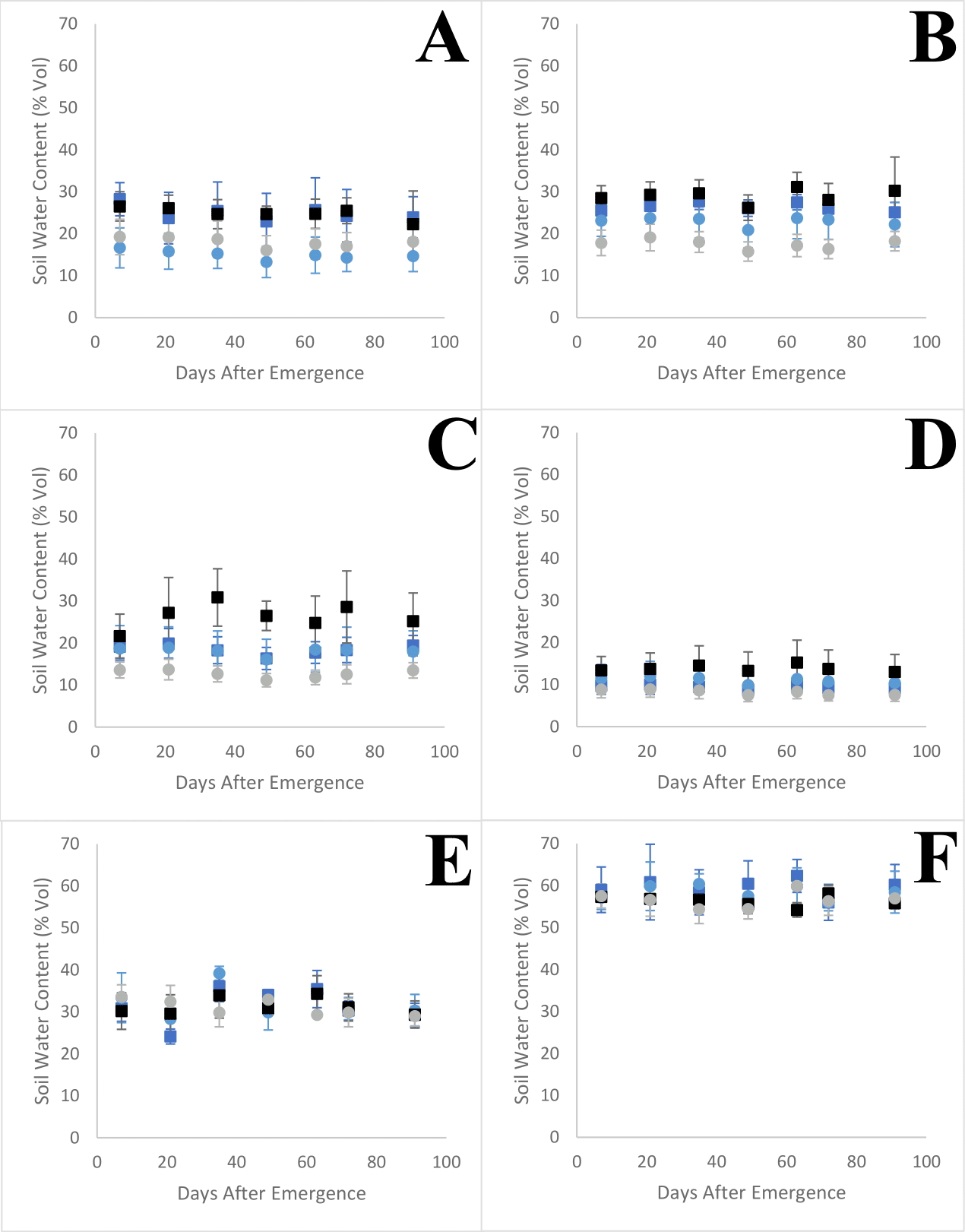
**

**Supplementary Figure 2.** Changes in soil water content over time in trial 2 at depths of 10 cm (A), 20 cm (B), 30 cm (C), 40 cm (D), 60 cm (E) and 100 cm (F). Each point represents the mean of three measurements of soil water content from either Inca Bella (blue) or Maris Piper (black/gray) plot with compacted (squares) or uncompacted (circles) treatment applied. Error bars indicate standard error with a p-value of 0.05.
